# Prevalence and associated factors of partially/non-immunization of under-five in Goma city, Democratic Republic of Congo: a community-based cross-sectional survey

**DOI:** 10.11604/pamj.2015.20.38.4483

**Published:** 2015-01-14

**Authors:** André Mwanishayi Kabudi, Prosper Mukobelwa Lutala, Junior Mulaja Kazadi, Inis Jane Bardella

**Affiliations:** 1 Docs Learning Centre, Département de Médecine de Famille Université de Goma, Quartier Keshyero Goma, Congo; 2 Department of Family Medicine, School of Public Health & Family Medicine, College of Medicine, University of Malawi; 3 Rosalind Franklin University of Medicine and Science Office of Global Health Initiatives, Department of Family and Preventive Medicine, Chicago Medical School, North Chicago, IL USA

**Keywords:** Incomplete immunization, determinants, children, Congo, Goma

## Abstract

**Introduction:**

At the East part of DRC, anecdotal reports are advancing several causes of unsuccessful campaigns of vaccination by the time going: rumors about use of vaccines for killing purpose, injection of vaccine to decrease the reproductive potential in coming generations, use of vaccines by some rebellions and neighboring countries to kill children indirectly, ineffectiveness of vaccines currently on the market. While those rumors seem to be less reliable, potential beneficiaries are taking them seriously and justifying a reluctance to bring their children or siblings for immunization. Against this above background, our community Primary Health Care team indicates that still, in Goma city in general and even in the referral hospital catchment area, there are children who have never been vaccinated.

**Objectives:**

To determine the prevalence and determinants of non-immunization of under-five children in Goma City.

**Study design:**

Cross-sectional community-based survey.

**Methods:**

A sample size of 384 children aged under-five years from the target population was used for the study. The ratio of under-five years of age Goma city to the total population of Goma city for the year 2012 was considered as the study population frame.

**Results:**

The prevalence of under-five non-immunized children was 25.7%. There was an association between immunization status of children and their gender, school characteristics, age, sibling, the level of literacy, the marital status of their parents and the age of their mothers.

**Conclusion:**

For improving the quality of under-five children immunization, the medical authorities must consider these different determinants.

## Introduction

One of the most important medical development in the twentieth century has been the control of once common childhood infectious diseases by the administration of highly effective vaccines [1]. Immunization saves lives of under-five children globally and more in developing countries. Every year, 10.6 million children die before the age of five years; 1.4 million of these are due to diseases that could have been prevented by vaccines. Taking into account both children and adults, vaccine-preventable diseases kill 3 million people around the world every year [2]. Globally, each year 130 million children are born, 91 million of which are in the developing countries. However, around 10 million children under the age of five years die every year and over 27 million infants in the world do not get full routine immunization. The estimate for global child deaths under five years was 10.8 million in 2000. About 41% of these were in Sub-Saharan Africa and 34% in South Asia. Immunization being one of the most Cost-effective public health interventions which is directly or indirectly responsible to prevent the bulk of mortalities in under-fives [3], it is wise to look at its uptake carefully, to draw lessons and address them accordingly. Immunization saves lives of under-five children globally and more in developing countries. The ultimate outcomes after immunization can either death or survival for children receiving it. However, even those who survive, serious complications and even death can occur thereafter. During the Millennium Development Goals meeting in 1990, the signatories agreed to curb the mortality rates of under-five due to preventable diseases by two-thirds by scaling up the immunization coverage in less industrialized countries. The past 2 decades have seen a radical detection of cases due to a global campaign against some childhood-preventable diseases namely diphtheria, measles, pertussis and polio [1]. Recently, WHO have reported a drastic reduction of deaths due to measles and its trends towards elimination. Even in Sub-Saharan Africa, some countries such as Rwanda, Malawi, Seychelles among others, mortality have been reduced drastically for their under-five unlike in others where mortality figures have shown a peak in new cases in recent years. In 2010 Congo was at the top with 72 029 reported deaths due to measles [4]. Locally in Goma, in spite of immunization campaigns conducted concurrently with the routine Immunization for close to a decade and aiming to catch up the few non-immunised children and therefore increase progressively the overall coverage of immunization, outcomes data from routine immunization campaigns in Goma are still alarming. Considering only measles as an indicator, in North-Kivu Province statistics showed that measles is still a public health problem. In 2012 for example, 489 cases of measles have been reported with 7 deaths (1.4 percent); while in 2013 the province reported 819 cases with 1 (0.1 percent) death [5]. If we can consider the low access to care in some villages and cities controlled by different armed groups, the difficulty of maintaining the cold chain for vaccines, malnutrition, over crowding in displaced population's camps, under reporting coming from all those problems, poor response of the health system to potential outbreaks,; some can easily understand the importance of scaling-up immunization coverage and ensuring close monitoring of immunization programs

The 189 signatory nations of the Millennium Development Goals (MDG) have committed to reduce mortality rates among children under five by two thirds between 1990 and 2015. Out of eight interrelated goals, MDG-4 regarding child mortality covers collective efforts of nations and technical agencies along with civil society organizations to scale up immunization coverage in less industrialized countries. There are numerous factors and barriers that hinder timely and effective immunization programs and adversely affect immunization coverage. These factors and barriers to immunization (Table 1) can be mainly grouped in three levels: (i) Individual & family level, (ii) society level and (iii) immunization program level 2 If many African countries have reached high immunization coverage, the situation is still bad in East of Congo with rates going as low as 40% due to conflicts and their after maths. Further armed conflicts and resurgence of diseases which were already declared eliminated, are two factors which are playing a role in funding recent immunization campaigns in the Democratic Republic of Congo (DRC) and some central-African countries [6]. A systematic education effort addressing misconception is needed. Physician, nurses and other providers of primary care have a unique opportunity to educate parents because parents see them as the most important source of information about immunization [7]. However, there is a need to tailor the message to local realities if a good response is expected. So far, few studies have addressed this gap. Before doing this systematic educational effort, we decided to assess the status of non-immunization of children among under-fives with the specific objectives to determine the prevalence of non-immunization in under-five children in the study area and secondly to determine the determinants of the non-immunization in those children

**Table 1:** Socio-demographic data of children

Variables	Frequence	percentage
**Sex of children**		
Males	195	59.6%
Females	132	40.4%
**Age ranges of children**		
< 6 months	7	2.1%
6 - 12 months	8	2.4%
1 – 5 years	312	95.4%
**Birthintervals**		
< 24 months	130	46.1
24–47 months	149	52.8
> 47 months	3	1.1
**Household'scharacteristics**		
Single parent	37	11.3%
Mixed parents	290	88.7%
**Distances betweenheathfacilities and houses**		
< 2 Km	327	100%
2–4 Km	0	0%
> 2 Km	0	0%
**Wealth index per month in USD**		
50-100	48	14.6%
101-150	49	14.9%
151-200	45	13.8%
201-250	43	13.1%
251-300	46	14.1%
301-350	10	3.1%
351-400	19	5.8%
401-450	9	2.8%
451-500	14	4.3%
501-550	3	0.9%
551-600	9	2.8%
651-700	3	0.9%
701-750	3	0.9%
951-1000	2	0.6%
>1000	14	4.3%
Unknown	10	3.1%

<:inferior; >: superior; Km: kilometers; USD: United States’ dollar; no participants indicated earnings in Wealth Indexes 601-650, 751-800, 801-900 and 901-950.

## Methods

**Study design:** This was a Cross-sectional community-based survey conducted among household in Goma health zone in which there was under-five children.

**Study setting:** The study was conduct in Goma city at North Kivu district, Democratic Republic of Congo designed to involve all under-five children. Routine immunization is organized in each health center one day per week by nurses who were in charge of immunization. The session of immunization begun with a health talk on a subject by a nurse. Mothers are divided in different groups according to the age of their children determining for most, the type of vaccine they will receive. Vaccines are provided by the government at an affordable cost. Mothers or guardians have to pay 900 Congolese Francs (equivalent to one United States dollar) to obtain an immunization card for their children during the first visit. At subsequent visits they pay200 Francs to receive the scheduled vaccine.

**Study population:** The study population was all under-five children living in Goma city. They were estimated at 127,516. Goma city had two municipalities: Karisimbi with seven quartiers and Goma with 6 quartiers. The ratio of the total number of rural quartiers to the total quartier of Goma city was 7/13= 0.538 and the ratio of the total number of urban quartiers to the total quartier of Goma city was 6/13= 0.461 Applying the above ratios within each municipality (due to the heterogeneous nature of the population of Goma city), a total of seven quartiers were selected, four of which were rural and three, urban. Within each municipality, rural and urban quartiers were selected randomly. First, we have randomly selected the quartier (the lowest administrative unit) from each commune, reflecting the urban/rural spread in each health zone, and with the number selected according to the population in each commune. The official list of communes provided by the Mayor's office was the sampling frame for the selection of quartiers. From each commune randomly selected two quartier (villages) in which 4 streets were further selected randomly. In each selected street, the sample included a group of 80 spreading out from a random starting point. They was no sampling within the site; all the eligible contiguous households up to 100 were included [8].

**Sampling size:** A sample size of 384 children aged under-five years from the target population was used for the study. The ratio of under-five years of age Goma city to the total population of Goma city for the year 2012 was considered as the study population frame. Bennett′s formula was used to calculate this sample size of 384 where Z is the z-score standard of 1.96, p is the expected prevalence of the group population (50% or 0.5), q = 1-p which is 0.5, d is the margin of error (degree of precision) which is 0.05 or 5%, deff is the design effect which is 1. Among the 384 children, 57 who had their immunization scheduling pending were excluded.

**Collection:** The data were collected by questionnaires in which there were questions regarding household characteristics and community profile. The researcher explained and administered questionnaires to mothers, once the consent was obtained. The immunization card was reviewed and cross-check immunization-related information.

**Data analysis:** The data were analyzed using SPSS (version 17.0) software to determine Pearson's Chi -Square and the likelihood ratio of categorical variables. The probability was set at 0.05 and the value was calculated to determine statistical significance of association. P-Values were used to determine whether differences were statistically significant

## Results

### Socio demographic data of participants

In Table 1 the number of male children is higher than females in our sample (59.6 versus 40.4 for females). Most children are greater than 12 months of age (95.4%), born less than four years after their siblings, and living in a household with both parents within a radius of two kilometers from the health facilities. Half of the parents of children were earning 0-250USD per month and only 4% earn 1,000USD per month.

### Socio-demographic data of parents

Households were predominately Christian in faith (91.1%) with parents married (85.9%). Most mothers were under the age of 31 (74%) and had completed a minimum of secondary school (63.9%), but were unemployed (57%). Most fathers had also completed a minimum of secondary school (82%) and were employed (85.6%). (Table 2)

**Table 2:** Sociodemographic data of parents

Variables	Frequence	Percentage
**Age of mothers**		
19-22	54	16.5%
23-26	113	34.6%
27-30	75	22.9%
31-34	25	7.6%
>34	58	17.7%
Unknown	2	0.6%
**Religion affiliation of parents**		
Christians	298	91.1%
Muslims	22	6.7%
No religion	7	2.2%
**Father's literacy**		
Primary school	39	11.9%
Secondary school	127	38.8%
University/tertiary	141	43.2%
Missing	20	6.1%
**Mother's literacy**		
Primary school	118	36.1%
Secondary school	142	43.4%
University	67	20.5%
**Marital status of mothers**		
Single	33	10.1%
Married	281	85.9%
Divorced	8	2.5%
Separated	5	1.5%
**Mother's occupation**		
Public sector	12	3.6%
Private sector	12	3.6%
Informal sector	100	30.6%
Students	17	5.2%
Unemployed	186	57%
**Father's occupation**		
Public sector	69	21.1%
Private sector	74	22.6%
Informal sector	137	41.9%
Students	14	4.3%
Unemployed	21	6.4%
Missing values system	12	3.7%

84 children did not receive any immunization, No religion means those without any affiliation or those who refused to give their religion for personal reasons.

### Immunization status

The overall prevalence of under-five incomplete immunization was 25.7% with the detail per vaccine in Table 3. All incompletely immunized children did not receive measles and yellow fever vaccines. However, for the remaining vaccines, only 5.6% of participants did not receive BCG, Polio, and DTC+Hib+PCV 13. Negligence of parents was the main cause of incomplete immunization. Among the children who were incompletely immunized, 69.0% were males against 31.0% of females.

**Table 3:** Immunization status

Variables	Frequence	Percentage
**Status**		
Incomplete	84	25.7%
Complete	243	74.3%
**Vaccines not received**		
BCG	5	5.6%
Polio 0	5	5.6%
Polio 1	5	5.6%
Polio 2	5	5.6%
Polio 3	5	5.6%
DTC+Hib+PCV13	5	5.6%
DTC+Hib+PCV13	5	5.6%
DTC+Hib+PCV13	5	5.6%
Measles	84	100%
Yellow fever	84	100%
**Reasons for non-immunization**		
Side effects	1	0.3%
Parental negligence	79	24.1%
Without importance	2	0.6%
Difficult access	2	0.6%

BCG: bacillus Calmette – Guerin vaccine, DTC: diphtérie-tétanos-coqueluche, PCV: pneumococcalconjugate vaccine, Hib: antiHaemophilus influenzae type b (Hib) vaccine. Reasons for non-immunizations proportions were calculated out of total participants.

### Determinants of full immunization

Table 4 details several statistically significant associations. Female children (Pearson Chi-Square= 4.162; p< 0.005), pre-school children (Pearson Chi-Square= 15.545; p< 0.01), and those greater than one year of age (Pearson Chi-Square= 22.061; p< 0.01) were more likely to be fully immunized. There were statistically significant associations between immunization status and parental demographics. For mothers, lower levels of education had higher rates of complete immunization (Pearson Chi-Square= 34.517; p< 0.01) whereas for fathers higher levels of education resulted in higher rates of immunization (Pearson Chi-Square= 28.391; p< 0.01). Divorced mothers, married mothers and mothers between the ages of 31 and 34 had children with the highest rates of complete immunization (Pearson Chi-Square= ***needs recalcuated***; p< 0.05 and Pearson Chi-Square= 28.432; p < 0.01), increasing of literacy level of their mother (Pearson Chi-Square= 34.517; p< 0.01), marital status (married) of parents (Likelihood ratio: 0.039; p< 0.05); and age ranges of mother (likelihood ratio= 28.432; p< 0.01). The chance to be completely immunized decreased for last born with the increasing of child number in the household. The location sibling and the immunization status was strongly associated (likelihood ratio was 28.157; p<0.01).

**Table 4:** Determinants of full immunization

Variables	IImmunization status				
	Incomplete N (%)	Complete N (%)	Total N (%)	Chi-square/ LL R	p-Value
**Sex of children**					
Males	58(69%)	137(56.4%)	195(59.6%)	4.162	<.005
Females	26(31%)	106(43.6%)	132(40.4%)		
**Schoolchildren**					
Preschool	3(3.6%)	55(22.6%)	58(17.7%)	15.545	<0.0001
No schooling	81(96.4%)	188(77.4%)	269(82.3%)		
**Children'sage**					
< 6 months	7(8.3%)	0(0%)	7(2.1%)	22.061	<0.01
6-9 months	4(4.8%)	4(1.6%)	8(2.4%)		
>1 year	73(86.9%)	239(88.4%)	312(95.5%)		
**Mother'seducation**					
Primary	7(8.3%)	70(59.3%)	77(23.5%)	34.517	<0.01
Secondary	4(4.8%)	107(75.4%)	111(34%)		
Tertiary/university	73(86.9%)	66(98.5%)	169(42.5%)		
**Father'seducation**					
Primary	21(53.8%)	18(46.2%)	39(12.7%)	28.391	<0.01
Secondary	37(29.1%)	90(90.9%)	127(41.4%)		
Tertiary/university	19(25.1%)	122(74.9%)	141(45.9%)		
**Marital status**					
Single	13(10.7%)	20(6.7%)	33(10.1%)	0.039	<0.05
Married	69(56.6%)	212(70.7%)	281(85.9%)		
Divorced	0(0%)	8(2.7%)	8(2.4)		
Separated	2(32.7%)	3(20%)	5(1.5)		
**Mothers’ age**					
19-22	24(44.4%)	30(55.6%)	54(16.5%)	28.432	<0.01
23-26	20(17.7%)	93(82.3%)	113(34.6%)		
27-30	13(17.3%)	62(82.7%)	75(23%)		
31-34	3(12%)	22 (88%)	25(7.6%)		
>34	22(37.9%)	36(62.1%)	58(17.7%)		
Unknown	2 (0.6%)	0 (0%)	2(0.6%)		

LLR:Likelihood-ratio test, N: frequency,%: percentage. For fathers’ education. Only 307 gave their answers, 20 were missing values as shown in Table 2.

### Child location in the siblings on immunization

Table 5 shows overall high immunization rates with no clear trend between immunization status and birth order in the family.. However, complete immunization is high for those born first, second and third of the families in respective 33.7, 24.7, and 17.3% des cas. When reaching the rank 4, 5, and 6; the proportions of incomplete immunization are high in respectively 9.5, 14.3 and 11.9% of cases. From rank 10 onward there is a slight high proportion of non-immunized children.

**Table 5:** Child location in the siblings

Sibling	Immunization status					
	Incomplete		Complete		Total	
	Frequence	Percentage	Frequence	Percentage	Frequence	Percentage
1	22	26.2%	82	33.7%	104	31.8%
2	16	19%	60	24.7%	76	23.2%
3	6	7.1%	42	17.3%	48	14.7%
4	8	9.5%	17	7%	25	7.6%
5	12	14.3%	16	6.6%	28	8.6%
6	10	11.9%	9	3.7%	19	5.8%
7	1	1.2%	6	2.5%	7	2.1%
8	2	2.4%	6	2.5%	8	2.4%
9	2	2.4%	2	0.8%	4	1.2%
10	1	1.2%	2	0.8%	3	0.9%
11	1	1.2%	0	0%	1	0.3%
12	2	2.4%	0	0%	2	0.6%
13	1	1.2%	1	0.4%	2	0.6%
Total	84	100%	243	100%	327	100%

Sibling: Rank in the sibling

## Discussion

The aim and objectives of this study conducted in Goma in the DRC were to determine the prevalence and determinants of incomplete immunization among under-five children in Goma. The prevalence of incomplete immunization among those children is 25.7% (Table 3). Male children, those with no schooling, and children less than one year of age had higher rates of incomplete immunization. Incomplete immunization was more prevalent for children whose fathers had not completed secondary school, whose mothers had university education, whose mothers were single or separated, and whose mothers were less than 23 years of age, Table 4.

Our age and gender findings are similar to those in the Khartoum and Bangladesh studies [8, 9]. Our association between immunization status and school characteristics can be explained by the fact that, our immunization schedule plan is for completion at nine months of age. After this age, children can be immunized when there is a vaccination campaign which is generally organized against poliomyelitis or measles.

Our study varied from the findings of Kawakatsu et al [4], Ibnouft et al [8], Samali [10], Omutanyi and Mwanthi [11], and Kamau and Esamai [12] who all found improved immunization rates with increasing parental education. While prevalence of complete immunization increased with fathers level of education, our prevalence decreased when mothers had a university level of education. This variance maybe due to the small proportion of mothers with a university level of education compared to secondary and primary school in our study. Wealth Index of the household may also have negative influence in our study since half of the households had incomes less than or equal to 250 USD per month and mothers had to pay for an immunization card and each immunization.

In our study (Table 5), children who are living with both parents have more chance to be immunized than who are living only with their mother. This situation can be justified by the high number of children who are coming from married couples. Even if the likelihood ratio shows a relationship between immunization status of children and marital status of their parents, the small number of parents who are divorced and separated, and lower number of single mothers compared to married prevent a strong conclusion.

Our study shows a significant relationship between age of mother and immunization status. Children of young mothers, 19-22 year of age, had lower rates of complete immunization. This result is consistent with the studies done in Kenya by Kawakatsu [4] and Omutanyi [11]. Age is associated with maturity. The maturity of the mother could explain their awareness about immunization. We observed in our study a tendency of less children completely immunized for mothers greater than 34 years olds. That could be explained by the small number of mothers in this age interval.

This study must be understood in light of several weaknesses. While this study has focused on a topic which is an epicenter of health authorities locally and internationally, Congo is at the top of the list of countries in the WHO-African region where cases of measles are still being reported. As of 2010, a drop of cases has been found globally (from 562,000 in 2000 to 122,000 in 2012), however DRC reported 72,029 of the 2012 cases. Measles is one of the markers of infant mortality which can give an idea on the decline and achievement of the Millennium Development Goals for DRC. Unfortunately immunization, which is one of the pillars in fighting childhood mortality, remains very poor in DRC with weaknesses in the whole health system. The main weakness of the study is its inability to document the immunization prevalence of some groups of children, like those born at home and not declared at a health facility. Parents’ concerns about the quality of vaccines used for immunization were not assessed by our questionnaire. The risk of recall bias for some information from mothers who did not bring the immunization cards and who were obliged to give information from memory was high.

## Conclusion

The present study indicates that the prevalence of incomplete immunization for under-five children in Goma is 25.7%. The determinants of under-five incomplete immunization in children are child's gender, child's school status, parent's level of education, age of the child, marital states of the parents, and the age of the mother.
